# Pyogenic spondylitis and paravertebral abscess caused by *Salmonella* Saintpaul in an immunocompetent 13-year-old child: a case report

**DOI:** 10.1186/s12887-018-1010-5

**Published:** 2018-02-03

**Authors:** Shota Myojin, Naohiro Kamiyoshi, Masaaki Kugo

**Affiliations:** Department of Pediatrics, Japanese Red Cross Society Himeji Hospital, 1-12-1, Shimoteno, Himeji, Hyogo 670-8540 Japan

**Keywords:** *Salmonella* Saintpaul, Pyogenic spondylitis, Paravertebral abscess, Psoas abscess, CT-guided biopsy

## Abstract

**Background:**

Salmonella spondylitis is an uncommon complication of Salmonella infection in immunocompetent children. To prevent treatment failure and neurological deficits, it needs prompt diagnosis and sufficient effort to identify the causative organism. There are some options to identify the causative organism such as Computed Tomography (CT) guided biopsy or surgical debridement, however when to perform these invasive interventions remains controversial.

**Case presentation:**

A 13-year-old boy presented with occasional high fever and lower back pain. He was diagnosed with spondylitis of the L4–5 vertebral bodies and paravertebral abscess. Initial blood cultures were negative, therefore empirical antibiotic treatment was started. He responded well to conservative management, and was discharged after clinical improvement. However, he was re-hospitalized 2 weeks after discharge, and surgical debridement was performed which led to the detection of *Salmonella* Saintpaul as the causative pathogen. It was revealed that the possible source of infection was consumption of raw poultry eggs, or contact with poultry. Definitive antibiotic therapy was started. He was discharged with good recovery after a 6-week hospitalization.

**Conclusions:**

This is the very first case report of pyogenic spondylitis caused by *Salmonella* Saintpaul. Salmonella should be considered as a causative pathogen of pyogenic spondylitis in immunocompetent children. Identifying the causative organism is essential to prevent treatment failure, and a high index of suspicion is needed for prompt diagnosis especially when blood cultures are negative. Invasive interventions such as CT-guided biopsy should be considered even if the clinical course seems to be uncomplicated.

## Background

Pyogenic spondylitis is a rare disease in immunocompetent children, and the exact incidence is still unclear due to the few quality case series available in the literature. The average age of diagnosis in children is approximately 2 to 8 years, and the incidence of involvement of the lumbar or lumbo-sacral region represents the majority of the cases although any level of the spine can be affected [1]. A wide range of organisms have been associated with spondylodiscitis. *Mycobacterium tuberculosis* is the commonest cause of spinal infection worldwide, and accounts for 9–46% of cases in developed countries [2]. The other organisms which can cause spondylodiscitis are *Staphylococcus aureus*, *Escherichia coli*, Pseudomonas, Streptococci, and Klebsiella [2]. Salmonellae are well known as organisms which cause a number of characteristic clinical infections in humans from gastroenteritis, enteric fever, and bacteremia to the asymptomatic carrier state. Focal metastatic infections such as osteomyelitis or abscess can occur, but they are extremely rare in immunocompetent children. It has been reported that Salmonella osteomyelitis constitutes 0.8% of all Salmonella infection, and only 0.45% of all types of osteomyelitis [3]. Precisely because spondylitis is uncommon in previously healthy children, it requires clinical suspicion for prompt diagnosis and sufficient effort including invasive interventions to identify the causative organisms especially when blood cultures are negative in order to prevent treatment failure.

We report a case of pyogenic spondylitis and paravertebral abscess caused by *Salmonella* Saintpaul in a previously healthy 13-year-old child, who required surgical interventions after clinical improvement by conservative treatment.

## Case presentation

A 13-year-old boy with no significant past medical history presented to our outpatient clinic due to back pain with fever. He had been well until approximately 4 months before admission, when he occasionally had high fever. He started to complain of the lower back pain during 3 months before admission, but he thought it was caused by his daily training of track and field. 4 days before admission, high fever developed. His back pain became so severe 3 days ago that he was seen by his family doctor. His symptoms did not improve in spite of acetaminophen administration.

His past medical history was unremarkable, without any trauma, surgical history, or recurrent bacterial infections. He was a junior high school student, and a track and field athlete. He always trained by himself around the corner of zoo, and had no contact history with animal. The patient had raw poultry eggs which were directly purchased from a neighborhood farm just a few weeks before he started complaining of occasional high fever.

On admission, the patient was conscious complaining of severe lumbar pain which was exacerbated according to any movement, and it was hard for him to walk without support due to the severe pain. On physical examination his vital signs were normal except a body temperature of 38 °C. Neurological examination demonstrated no motor or sensory deficits, and other physical findings were nonspecific, and any gastrointestinal signs were not identified.

**Fig. 1:**
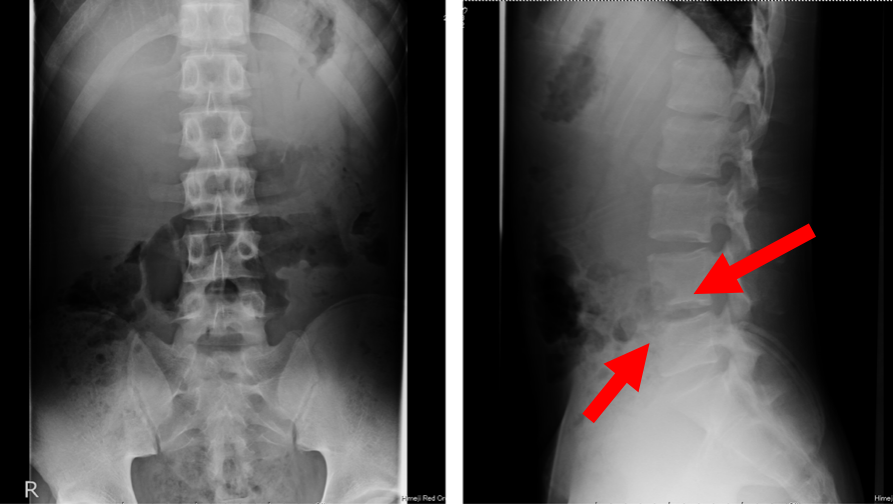
X-ray revealed inhomogeneous appearance of the inferior wall of the L4 and anterior wall of the L5 vertebral bodies

**Fig. 2:**
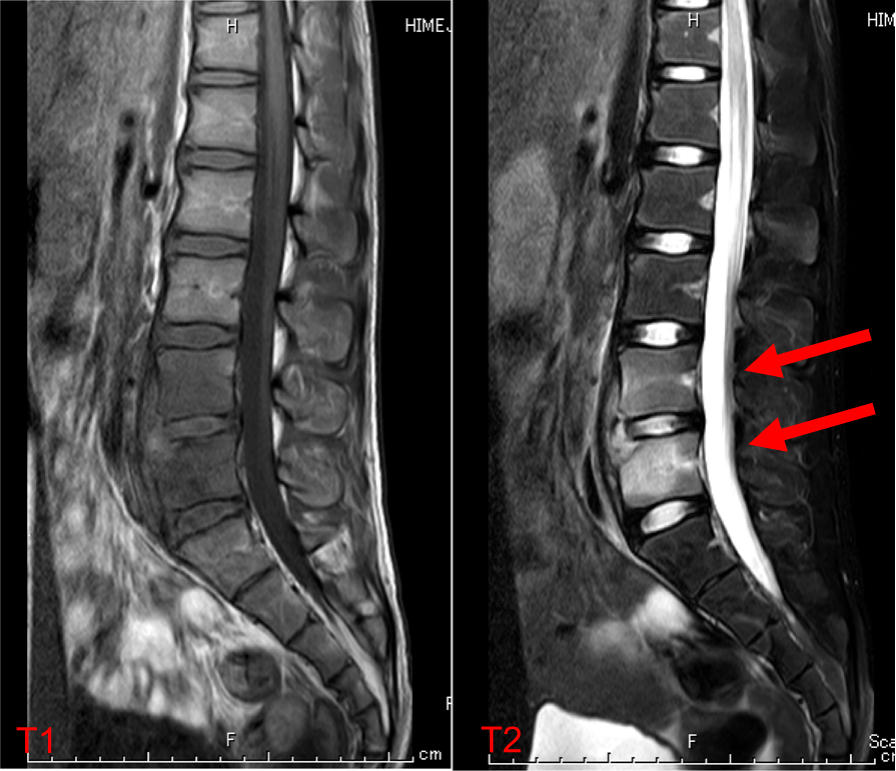
MRI on the second day of hospitalization. Abnormal hyperintensity of vertebral bodies of L4 and L5 surrounded by paravertebral soft tissue low diffusion. The interior of the spinal canal was intact

Laboratory exams showed a slight increase in white blood cell count (WBC 10,100 /μL; neutrophils 61%, lymphocytes 29%) and elevation of C-reactive protein (CRP 7.27 mg/dL). Initial blood cultures were negative. His immunoglobulin G, A, M level were within normal range. Interferon-γ based release assay (QuantiFERON-TB GOLD) was negative. Chest X-ray and abdominal ultrasound showed no abnormal findings. The lumbar lateral radiologic findings showed inhomogeneous appearance of the inferior wall of the L4 and anterior wall of the L5 vertebral bodies (Fig. 1). Thoracic and lumbar magnetic resonance imaging (MRI) showed abnormal high signal of the vertebral bodies of L4–5 in sequences of T2-weighted images and paravertebral low diffusion in sequences of T1-weighted images. The interior of the vertebral canal was intact (Fig. 2).

**Fig. 3:**
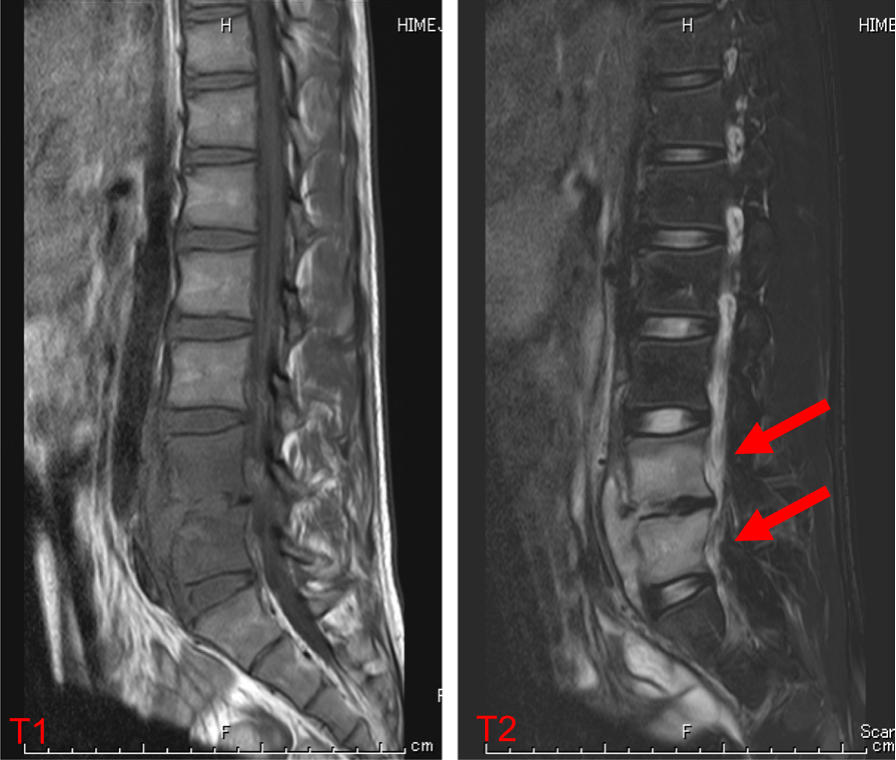
MRI on the day of re-hospitalization showed exacerbation of vertebral disc destruction. Abnormal hyperintensity of vertebral bodies of L4 and L5, and paravertebral soft tissue low diffusion are also seemingly worse than Fig. 2

**Fig. 4:**
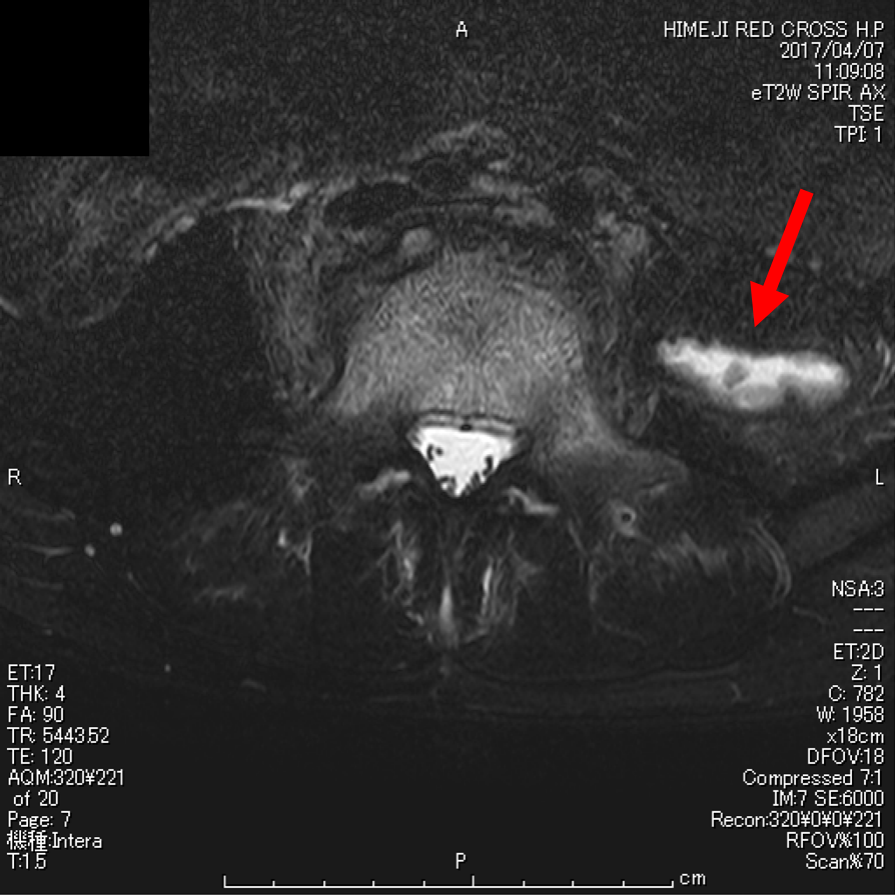
MRI after 2 weeks showed newly diagnosed left psoas abscess

**Fig. 5:**
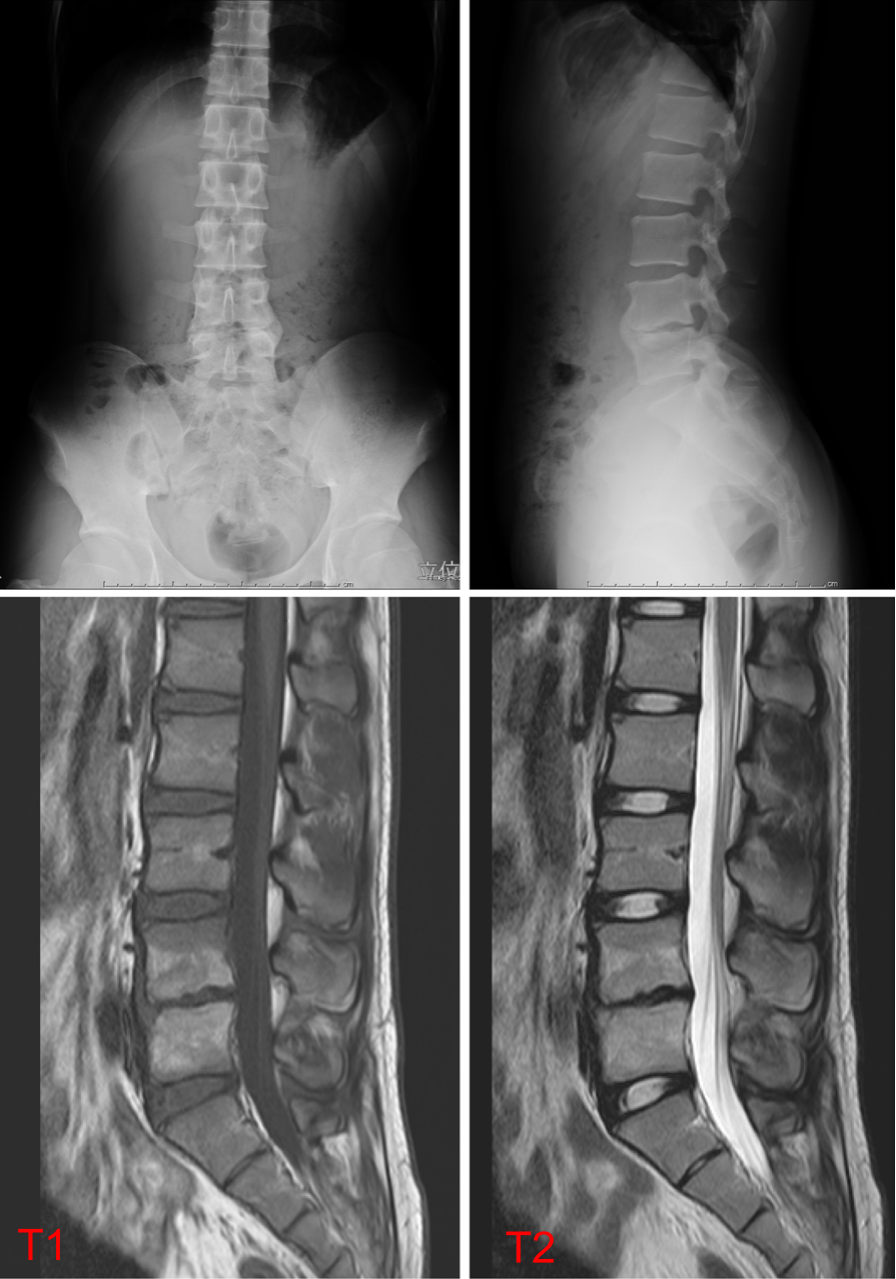
The radiograph and MRI at the point of 6 months follow-up after discharge revealed improvement of vertebral bodies alignment, and no exacerbation of abscess formation or bone destruction

The patient was diagnosed as pyogenic spondylitis and paravertebral abscess. We began antibiotic therapy empirically using cefazolin and clindamycin to cover *Staphylococcus aureus* and Gram-negative organisms. The patient remained febrile and CRP was elevating even after starting the initial antibiotic therapy, therefore we switched the antibiotic to vancomycin assuming community acquired methicillin-resistant *Staphylococcus aureus* (MRSA) as the possible causative organism. Soon after starting vancomycin the patients fever reduced and his CRP returned to the normal range. He remained afebrile during the 3-week administration of vancomycin, so we switched the antibiotic to oral linezolid. He was discharged after we made sure that he remained afebrile and CRP was negative for a week. During this course, we did not perform percutaneous CT-guided biopsy since it seemed to be quite invasive given the location of inflammation and abscess, and the clinical course was favorable. However, he began exacerbation of back pain 2 weeks later after discharge, and was hospitalized again. The laboratory test showed the elevation of CRP, and MRI showed major destruction of the vertebral bodies (Fig. 3). To prevent neurological deficits due to treatment failure, we stopped administering linezolid and performed surgical drainage, and transplantation of iliac crest graft following curettage of the vertebral disc. During and after the surgery, we used sulbactam cefoperazone empirically. Tissue, wound and abscess cultures from the surgical specimens grew *Salmonella* Saintpaul which was sensitive to cefotaxime, therefore we changed the antibiotic to cefotaxime. His fever reduced and CRP began to decline soon after the surgery, but 2 weeks after starting cefotaxime, follow-up MRI showed a left sided psoas abscess (Fig. 4). We performed CT-guided biopsy and debridement, which led to no exacerbation in symptoms and laboratory data afterward. Cefotaxime was administered for a total of 4 weeks, and after making sure that he remained afebrile and CRP remained within the normal range, we switched the antibiotics to oral trimethoprim-sulfamethoxazole which the organism was susceptible to. He was discharged and finished taking trimethoprim-sulfamethoxazole for a total of 2 weeks. The radiograph and MRI at the point of 6 months follow-up after discharge revealed improvement of vertebral bodies alignment, and no exacerbation of abscess formation or bone destruction (Fig. 5). He currently shows no neurological problems, and is under follow-up observation every 2 to 3 month at our outpatient clinic with good recovery.

## Discussion

This is the first report of pyogenic spondylitis caused by *Salmonella* Saintpaul. Pyogenic spondylitis is a known, but relatively rare complication of Salmonella infection. In two recent reviews of spondylodiscitis in children, positive blood cultures were obtained respectively in 7 out of 16 and 1 out of 18 children, however none were affected by Salmonella [4, 5]. In immunologically normal children, this infection is a rare condition and is mainly reported in case reports. The 4 most common strains of Salmonella causing osteomyelitis in adults are *Salmonella* Typhimurium, *Salmonella* Enteritidis, *Salmonella enterica* subsp. Arizonae and *Salmonella* Typhi [3]. There are a few reports of pediatric vertebral infection in which the strains of Salmonella could be successfully identified including *Salmonella* Oranienburg [6], *Salmonella* Agona [7], *Salmonella* Enteritidis and *Salmonella* Corvallis [3], however we could not find reports of spondylodiscitis caused by *Salmonella* Saintpaul. The principal reservoirs for nontyphoidal Salmonella organisms include birds, mammals, reptiles, and amphibians, and the major food vehicles of transmission to humans in industrialized countries include food of animal origin, such as poultry, beef, eggs, and the other food contaminated by contact with an infected animal product or a human carrier [8]. There were some outbreaks reports of *Salmonella* Saintpaul gastroenteritis caused by environmental contamination of food or drink [9, 10]. In Japan, the most prevalent serotype in human salmonellosis is Enteritidis, and it is often associated with contaminated eggs [11]. There are 3 Japanese case reports of spondylodiscitis caused by Salmonella in immunocompetent children [6, 7, 12], and in one case it was strongly suspected that consumption of a dried squid product was associated with the infection course [7]. From these bacterial characteristics, it is important to take a detailed social history including dietary history, and life environment in order to identify the causative organism.

Our patient was an immunocompetent child without any medical history, therefore why he had gotten Salmonella spondylitis was inexplicable. After the detection of *Salmonella* Saintpaul from the surgical specimens, we took a thorough medical history again to find the route of infection. He might have had chronic small injuries because he was a track and field athlete, but he denied any recent traumatic wounds or fractures which could cause contiguous infection to the vertebrae. *Salmonella* Saintpaul has never been reported to cause a chronic carrier state, therefore it is unlikely that the patient was a chronic carrier of the pathogen. The only clues to the source of infection were the following two. First, he had raw poultry eggs which were directly purchased from a neighborhood farm just a few weeks before he started complaining of occasional high fever. He had raw eggs on rice (Tamago-Kake-Gohan, in Japanese) for breakfast occasionally, and we thought this was the probable event which caused hematogenous spread of the pathogen to the vertebrae. In Japan, we have a tradition of having raw eggs, and there are reports of severe Salmonella infections including osteomyelitis as sequelae of raw egg consumption [13, 14]. Secondly, he might have had contact with poultry and its feces. The athletic field where he always trained was close to the zoo. Animal houses, such as chickens, natatorial birds and peacocks were adjacent to the training course. He also mentioned that he always took a rest at the point which was surrounded by bird houses. Therefore, it was possible that he inhaled or contacted small amounts of poultry feces. We concluded that our patient had gotten *Salmonella* Saintpaul infection by consumption of raw poultry eggs or contact with poultry feces, which possibly caused secondary bacteremia and hematogenous spread of the pathogen to the vertebrae.

Although childhood spondylodiscitis is thought to be benign and self-limiting, some cases have residual neurological sequelae. Therefore, it is essential to diagnose promptly, identify the causative organism, and start definitive therapy as soon as possible in order to prevent treatment failure. A high index of suspicion is needed for prompt diagnosis, and it requires sufficient effort to identify the causative organism especially when the blood cultures are negative. Some authors advocate that antimicrobial treatment should not be started until the organism has been identified except in patients who are at risk, for instance, those with neutropenia or severe sepsis [2]. When the blood cultures are positive, the causative organism is easily suspected since the infection is mostly monomicrobial and often has a hematogenous source. It is reported that the yield from blood cultures varies between 40% and 60% in clinically defined cases of pyogenic spondylodiscitis [2]. There is another option to identify the causative organism especially when blood cultures are negative: CT-guided biopsy. It is generally recommended to perform CT-guided biopsy when the response to initial conservative therapy is not good, and atypical organisms are suspected as causative pathogens [2]. Some authors advocate that it should be reserved for cases that do not respond to initial empirical therapy [15]. Given its invasiveness, we thought it was plausible that empirical therapy should be initiated based on the assessment of the probable organisms. The most frequent causative organisms of spondylitis are Staphylococci and Streptococci, and there are also reports of gram-negative, low virulent and atypical organisms isolated. Therefore, the recommended initial antibiotics are a combination of third generation cephalosporins and oxacillin / clindamycin [16]. We chose cefazolin and clindamycin with a strong suspicion of methicillin-sensitive *Staphylococcus aureus* (MSSA) as the causative organism. Since the initial treatment did not result in clinical improvement, we changed the antibiotics to vancomycin to cover community acquired MRSA. The patient showed drastic improvement both in symptoms and laboratory data soon after starting vancomycin. This is the principal reason why we did not perform CT-guided needle biopsy despite all the blood cultures being negative and the causative organism not being identified. It’s no wonder that vancomycin or linezolid does not have much effect on Salmonella as they are mainly used to treat gram positive cocci infections. However, it is possible to speculate that intravenous administration of vancomycin monitored strictly by therapeutic drug monitoring (TDM) was more effective than oral linezolid in this case. Since it is important to make sure that the antibiotic remains at a high enough concentration at the focus of infection to treat osteomyelitis, switching to oral linezolid at a normal dose might be the reason why his clinical condition relapsed after discharge. Since we were concerned about treatment failure, we decided to perform surgical debridement to identify the causative organism and its sensitivity to antibiotics. As a result, we could successfully identify *Salmonella* Saintpaul as the causative organism, and start the most effective antibiotic therapy leading to clinical improvement and no neurological impairment. It is reported that childhood spondylodiscitis has a generally good prognosis, but disability due to residual neurological deficit or severe pain can occur as a sequelae of treatment failure. In as many as 20% of children functional deficits were present in a German retrospective study [15]. In a reported series, which included 42 patients, three out of them had pain when exercising, and one patient had long-term neurological sequelae [17]. Fortunately, our patient shows no pain or neurological deficits currently, however it goes without saying that starting the definitive therapy as soon as possible improves the prognosis and reduces the length of the period of hospitalization. Therefore, invasive interventions including CT-guided biopsy and surgical drainage should be considered to identify the causative organism especially when it remains unknown.

## Conclusion

Salmonella can cause spondylitis in previously healthy immunocompetent children, therefore it should be considered as a causative pathogen. Identifying the causative organism is essential to prevent treatment failure. CT-guided needle biopsy or other surgical interventions should be considered in order to identify the causative organism especially when blood cultures are negative, even if the clinical course seems to be promising. A detailed social history can help find the infection route.

## Abbreviations

CRP: C-reactive protein
CT: Computed tomography
MRI: Magnetic resonance imaging
MRSA: Methicillin-resistant *Staphylococcus aureus*
MSSA: Methicillin-sensitive *Staphylococcus aureus*
TDM: Therapeutic drug monitoring
WBC: White blood cell

## References

[1] Fucs PM, Meves R, Yamada HH (2012). Spinal infections in children: a review. Int Orthop.

[2] Gouliouris T, Aliyu SH, Brown NM (2010). Spondylodiscitis: update on diagnosis and management. J Antimicrob Chemother.

[3] Tsagris V, Vliora C, Mihelarakis I, Syridou G, Pasparakis D, Lebessi E, Tsolia M (2016). Salmonella Osteomyelitis in previously healthy children: report of 4 cases and review of the literature. Pediatr Infect Dis J.

[4] Chandrasenan J, Klezl Z, Bommireddy R, Calthorpe D (2011). Spondylodiscitis in children: a retrospective series. J Bone Joint Surg Br.

[5] Tapia Moreno R, Espinosa Fernández MG, Martínez León MI, González Gómez JM, Moreno Pascual P (2009). Spondylodiscitis: diagnosis and medium-long term follow up of 18 cases. An Pediatr (Barc).

[6] Matsumoto M, Mori R, Kinoshita G, Maruoka T, Maruo S (2000). Salmonella vertebral osteomyelitis. J Lumbar Spine Disord.

[7] Ishigami S, Yoshida M, Kawakami M, Hashizume H, Nakagawa Y, Kioka M (2007). Vertebral Osteomyelitis caused by salmonella Agona. Clin Orthop Surg.

[8] Kimberlin DW, Brady MT, Jackson MA, Long SS. Salmonella Infections. In: Red Book 2015, Report of the Committee on Infectious Diseases. 30th ed. American Academy of Pediatrics. p. 695–702.

[9] Draper AD, Morton CN, Heath JN, Lim JA, Markey PG (2017). An outbreak of *Salmonella* Saintpaul gastroenteritis after attending a school camp in the northern territory. Australia Commun Dis Intell Q Rep.

[10] Centers for Disease Control and Prevention (CDC). Multistate Outbreak of *Salmonella* Saintpaul Infections Linked to Imported Cucumbers (Final Update). https://www.cdc.gov/Salmonella/saintpaul-04-13/index.html. Accessed 27 Jan 2018.

[11] National Institute of Infectious Diseases (NIID) (2009). Salmonellosis in Japan as of June 2000. IASR Infect Agents Surveillance Rep.

[12] Dohi O, Ito K, Takamatsu K, Takahashi N, Aikawa J (2006). A case report of salmonella spondylitis in child. Tohoku J Orthop Traumatol.

[13] Matsubara K, Tabara S, Katayama T, Nishi H, Haritani H, Yura K (2003). Salmonella enteritidis Osteomyelitis of the tibia - a case report and review of literature on salmonella Osteomyelitis of Japanese patients. JJAInfD.

[14] Ishikawa J, Yamamuro M, Togawa M, Shiomi M (2009). Acute encephalopathy caused by salmonella enteritidis in a 9-year-old girl. J Pediatr Infect Dis Immunol.

[15] Kayser R, Mahlfeld K, Greulich M, Grasshoff H (2005). Spondylodiscitis in childhood: results of a long-term study. Spine.

[16] Waizy H, Heckel M, Seller K, Schroten H, Wild A (2007). Remodeling of the spine in spondylodiscitis of children at the age of 3 years or younger. Arch Orthop Trauma Surg.

[17] Garron E, Viehweger E, Launay F, Guillaume JM, Jouve JL, Bollini G (2002). Nontuberculous spondylodiscitis in children. J Pediatr Orthop.

